# Thermodynamic Study of the Complexation of *p*-Isopropylcalix[6]arene with Cs^+^ Cation in Dimethylsulfoxide-Acetonitrile Binary Media

**DOI:** 10.3390/molecules16098130

**Published:** 2011-09-22

**Authors:** Saeid Ahmadzadeh, Anuar Kassim, Majid Rezayi, Gholam Hossein Rounaghi

**Affiliations:** 1 Department of Chemistry, Faculty of Sciences, Universiti Putra Malaysia, 43400 Serdang, Selangor, Malaysia; Email: anuar@science.upm.edu.my (A.K.); chem_rezayi@yahoo.com (M.R.); 2 Department of Chemistry, Faculty of Sciences, Ferdowsi University of Mashhad, Mashhad, Iran; Email: ghrounaghi@yahoo.com (G.H.R.)

**Keywords:** conductometry, dimethylsulfoxide–acetonitrile binary media, *p*-isopropylcalix-[6]arene, Cs^+^ cation

## Abstract

The complexation reactions between the macrocyclic ionophore, *p*-isopropylcalix[6]arene and Cs^+^ cation were studied in dimethylsulfoxide–acetonitrile (DMSO-AN) binary non-aqueous solvents at different temperatures using a conductometry method. The conductance data show that the stoichiometry of the (*p*-isopropylcalix[6]-arene·Cs)^+^ complex in all binary mixed solvents is 1:1. The stability of the complexes is affected by the composition of the binary solvent media and a non-linear behavior was observed for changes of log K_f_ of the complex versus the composition of the binary mixed solvents. The thermodynamic parameters (Δ*H°_c_* and Δ*S°_c_*) for formation of (*p*-isopropyl-calix[6]arene·Cs)^+^ complex were obtained from temperature dependence of the stability constant and the obtained results show that the (*p*-isopropylcalix[6]arene·Cs)^+^ complex is enthalpy destabilized, but entropy stabilized, and the values of the mentioned parameters are affected strongly by the nature and composition of the binary mixed solvents.

## 1. Introduction

Since Pedersen synthesized in 1967 the first macrocyclic polyether, namely dibenzo-18-crown-6 (DB18C6), the chemistry of macrocyclic compounds and metal ions has gained great attention due to its significance in the chemical and biochemical fields. The existence of donor atoms such as oxygen and nitrogen enables macrocyclic compounds to form stable and selective complexes with alkali, alkaline earth and transition metal ions through electrostatic attractions and encapsulation into the macrocyclic cavity [1,2]. The study of various macrocyclic compounds in different solvents or solvent mixtures may indicate new approaches for developing pharmaceutical systems or as a way to cross the blood organ barrier.

Since various studies have been done on calixarenes, their complexation behavior and chemical structure are clearly understood [3]. Due to the presence of phenyl groups which can form semi-rigid cone shapes, calixarenes acquire a pre-organized structure. The selectivity of calixarenes can be enhanced by adding numerous substituents to its lower rim to increase the complexation behavior. Moreover, the three-dimensional structure of the binding site which has been proved to exist both in solid state [4] and in solution [5] results in more efficient complexation of cations.

To study the formation of complexes between macrocyclic compounds with different metal ions in solution, various physicochemical techniques such as spectrophotometry [6], polarography [7], NMR spectrometry [8], calorimetry [9], potentiometry [10] and conductometry [11,12] have been used. Among these various methods, the conductometric technique offers the advantages for such investigations of sensitive and accurate measurement as well as inexpensive cost with a simple experimental arrangement.

The enthalpy and entropy values of complexation reactions are influenced by the amount of cation-solvent, macrocyclic compounds-solvent, complex-solvent and even solvent-solvent interactions [13,14]. Owing to the extensive use of non-aqueous solvents in a wide range of pure and applied chemistry experiments, which have led to rapid progression in chemical science technologies, it was of interest to extend our study to the mixed binary non-aqueous solvents.

**Figure 1:**
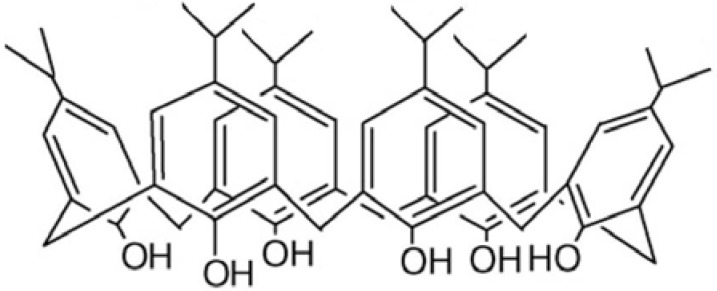
Chemical structure of *p*-isopropylcalix[6]arene.

In the current work, the complexation behavior of *p*-isopropylcalix[6]arene (Figure 1), toward cesium (I) ion was studied thermodynamically in dimethylsulfoxide–acetonitrile (DMSO-AN) binary non-aqueous solvents and the effect of solvent composition on thermodynamic parameters of complex formation was investigated at different temperatures using a conductometry method. Literature surveys show that there are no reports on the thermodynamic study of complex formation between *p*-isopropylcalix[6]arene with any metal cations.

## 2. Results

**Figure 2:**
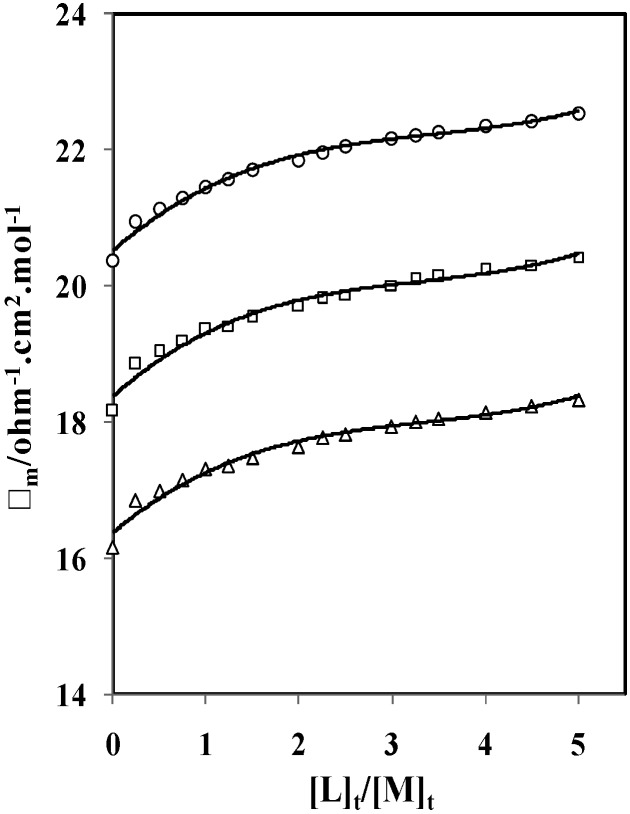
Molar conductance-mole ratio plots for (*p*-isopropylcalix[6]arene·Cs)^+^ complexes in DMSO-AN binary system (mol% DMSO = 15.5) at different temperatures (∆ = 25 °C, □ = 35 °C, ○ = 45).

Since the nature and composition of the solvent system can impressively affect the thermodynamic stability, stoichiometry, selectivity and exchange kinetics of cation-ionophore complexes, the changes of molar conductance (Λ*_m_*) versus the ionophore (*L*) to metal cation (*M*) mole ratio ([*L*]_t_/[*M*]_t_) for the complexation of *p*-isopropylcalix[6]arene with Cs^+^ cation in DMSO-AN binary systems were studied at different temperatures. [*L*]_t_ is the total concentration of the ionophore and [*M*]_t_ is the total concentration of the metal cation. One typical molar conductance values as a function of ([*p*-isopropylcalix[6]arene]/[Cs^+^]) mole ratio plots in DMSO-AN (mol% DMSO = 15.5) binary system is exhibited in Figure 2. The experimental data of molar conductance of *p*-isopropylcalix[6]arene-CsCl system in DMSO-AN binary media at different temperatures are listed in Table 1.

Since the ion-pair formation constant of an electrolyte can be written as below:

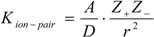
(1)
where A is constant, Z_+_ and Z_-_ are the charge of the electrolyte ions, r is the distance between the centers of the ions and D is the dielectric constant of the solvent.

In our case, since the charge of Cs^+^ and Cl^−^ is 1, the size of the ions, especially Cs^+^, is large (r, is large), the dielectric constant of the AN and DMSO are relatively high (*ε*: DMSO = 46.7, AN = 37.5), in addition, in the current solvent system the cation and anion are strongly solvated in solution and especially the concentration of the CsCl is very low, therefore, the ion pair association is negligible and consequently, the phenomenon of ionic association was not included in the current work.

**Figure 3:**
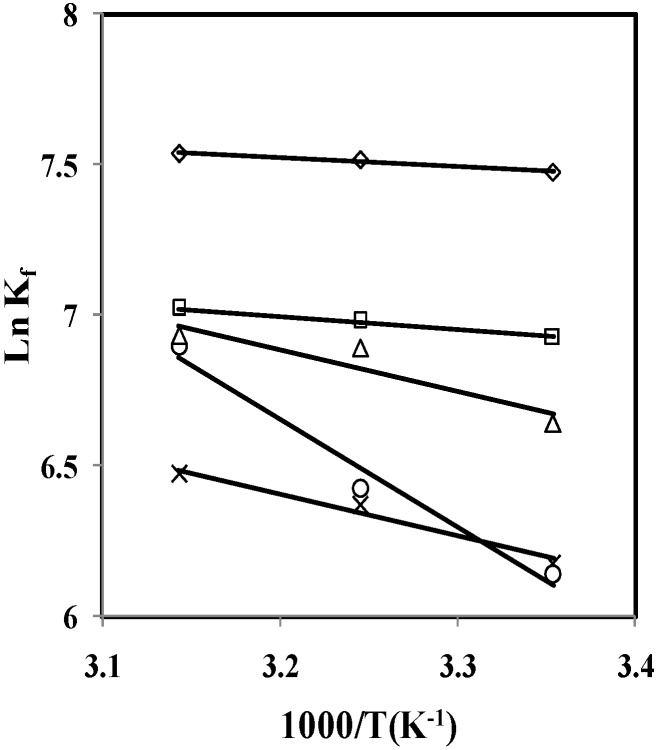
Van’t Hoff plots for (*p*-isopropylcalix[6]arene·Cs)^+^ complexes in DMSO-AN binary systems (mol% DMSO: ∆ = 100, ○ = 74.6, × = 52.5, □ = 32.9, ◊ = 15.5).

The stability constant for the formed complexes at each temperature was obtained by using non-linear least-squares GENPLOT computer program. The values of the stability constants (log K_f_) for the (*p*-isopropylcalix[6]arene·Cs)^+^ complex in various solvent systems are listed in Table 2. Plots of Ln K_f_ versus 1/T in all cases were linear, as shown in Figure 3.

**Table 1:** Molar conductivity of *p*-isopropylcalix[6]arene–CsCl system in DMSO-AN binary media at different temperatures.

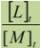	Λ *_m_*/ohm^−1^.cm^2^.mol^−1^														
	Pure DMSO			74.6% DMSO*			52.5% DMSO *			32.9% DMSO *			15.5% DMSO *		
	25 °C	35 °C	45 °C	25 °C	35 °C	45 °C	25 °C	35 °C	45 °C	25 °C	35 °C	45 °C	25 °C	35 °C	45 °C
**0**	**2.40**	**5.03**	**7.18**	**6.00**	**7.72**	**9.20**	**10.80**	**11.62**	**13.58**	**14.54**	**16.66**	**18.94**	**16.16**	**18.15**	**20.36**
**0.25**	**2.52**	**5.15**	**7.33**	**6.16**	**7.87**	**9.42**	**11.02**	**11.84**	**13.84**	**14.89**	**17.03**	**19.32**	**16.84**	**18.85**	**20.95**
**0.5**	**2.74**	**5.30**	**7.53**	**6.38**	**8.07**	**9.54**	**11.20**	**12.14**	**14.02**	**15.05**	**17.20**	**19.49**	**16.98**	**19.03**	**21.12**
**0.75**	**2.90**	**5.40**	**7.68**	**6.50**	**8.19**	**9.66**	**11.38**	**12.32**	**14.18**	**15.18**	**17.32**	**19.61**	**17.14**	**19.20**	**21.30**
**1**	**3.02**	**5.48**	**7.81**	**6.62**	**8.30**	**9.78**	**11.48**	**12.46**	**14.32**	**15.30**	**17.44**	**19.72**	**17.30**	**19.35**	**21.45**
**1.25**	**3.12**	**5.58**	**7.90**	**6.74**	**8.42**	**9.94**	**11.60**	**12.60**	**14.46**	**15.35**	**17.49**	**19.79**	**17.35**	**19.41**	**21.56**
**1.5**	**3.22**	**5.64**	**8.00**	**6.84**	**8.51**	**10.00**	**11.72**	**12.72**	**14.56**	**15.41**	**17.55**	**19.85**	**17.48**	**19.54**	**21.70**
**2**	**3.34**	**5.74**	**8.13**	**7.00**	**8.67**	**10.14**	**11.90**	**12.94**	**14.78**	**15.55**	**17.69**	**19.99**	**17.64**	**19.70**	**21.85**
**2.25**	**3.42**	**5.84**	**8.20**	**7.12**	**8.77**	**10.18**	**11.98**	**13.04**	**14.88**	**15.60**	**17.74**	**20.03**	**17.76**	**19.82**	**21.96**
**2.5**	**3.48**	**5.90**	**8.27**	**7.18**	**8.83**	**10.27**	**12.09**	**13.16**	**14.94**	**15.69**	**17.83**	**20.13**	**17.81**	**19.87**	**22.05**
**3**	**3.62**	**5.96**	**8.40**	**7.34**	**8.88**	**10.34**	**12.24**	**13.32**	**15.10**	**15.79**	**17.93**	**20.23**	**17.94**	**20.01**	**22.16**
**3.25**	**3.68**	**5.96**	**8.44**	**7.40**	**8.94**	**10.38**	**12.30**	**13.42**	**15.18**	**15.86**	**18.00**	**20.29**	**18.01**	**20.09**	**22.21**
**3.5**	**3.74**	**6.00**	**8.50**	**7.48**	**9.01**	**10.42**	**12.38**	**13.50**	**15.24**	**15.90**	**18.04**	**20.32**	**18.05**	**20.14**	**22.26**
**4**	**3.82**	**6.06**	**8.57**	**7.60**	**9.14**	**10.52**	**12.54**	**13.66**	**15.36**	**15.97**	**18.11**	**20.40**	**18.13**	**20.22**	**22.34**
**4.5**	**3.92**	**6.14**	**8.67**	**7.74**	**9.27**	**10.62**	**12.64**	**13.80**	**15.48**	**16.07**	**18.21**	**20.50**	**18.23**	**20.31**	**22.42**
**5**	**4.02**	**6.22**	**8.68**	**7.84**	**9.37**	**10.68**	**12.78**	**13.94**	**15.62**	**16.16**	**18.30**	**20.60**	**18.33**	**20.42**	**22.53**

* Composition of binary mixture is expressed in mol% for solvent system.

**Table 2:** *Log K_f_* values of (*p*-isopropylcalix[6]arene·Cs)^+^ complex in DMSO-AN binary mixed solvent at different temperatures.

	Complex		*Log K_f_ ± SD ^a^*		
	Solvent medium				
(*p*-isopropylcalix[6]arene·Cs)^+^ DMSO-AN *^b^*			25 °C	35 °C	45 °C
Pure AN			*c*	*c*	*c*
15.5%DMSO-84.5%AN			3.2 ± 0.1	3.3 ± 0.1	3.3 ± 0.1
32.9%DMSO-67.1%AN			3.0 ± 0.1	3.0 ± 0.1	3.1 ± 0.1
52.5%DMSO-47.5%AN			2.7 ± 0.1	2.8 ± 0.1	2.81 ± 0.04
74.6%DMSO-25.4%AN			2.7 ± 0.1	2.8 ± 0.1	3.0 ± 0.1
Pure DMSO			2.9 ± 0.1	3.0 ± 0.1	3.01 ± 0.04

*^a^ SD* = Standard deviation; *^b^* Composition of binary mixture is expressed in mol% for solvent system; *^c^* The salt is not dissolved.

**Figure 4:**
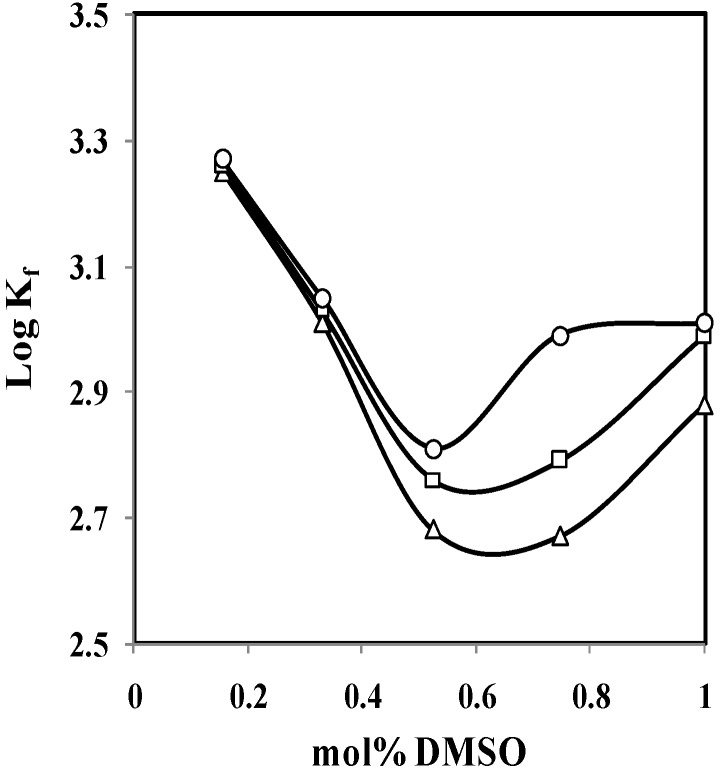
Changes of the stability constant (*log K_f_*) of (*p*-isopropylcalix[6]arene·Cs)^+^ complexes with the composition of DMSO-AN binary systems at different temperatures (∆ = 25 °C, □ = 35 °C, ○ = 45 °C).

The changes in standard enthalpy (Δ*H°_c_*) for complexation reactions were determined in the usual manner from the slope of the van’t Hoff plot by assuming that Δ*C_p_* is equal to zero over the entire temperature range investigated. The changes in standard entropy (ΔS°_c_) were calculated from the relationship Δ*G°_c,298.15_* = Δ*H°_c_* − 298.15 Δ*S°_c_*. The results are summarized in Table 3. The changes of log K_f_ of (*p*-isopropylcalix[6]arene·Cs)^+^ complex versus the mole fraction of dimethylsulfoxide in DMSO-AN binary system at different temperatures are demonstrated in Figure 4.

**Table 3:** Thermodynamic parameters for (*p*-isopropylcalix[6]arene·Cs)^+^ complex in DMSO-AN binary mixed solvent.

	Complex		Δ *G°_c_* ± *SD ^a^* (kJ.mol^−1^)	Δ *H°_c_ ± SD ^a^* (kJ.mol^−1^)	Δ *S°_c_* ± *SD ^a^* (J. mol^−1^ K^−1^)
	Solvent medium				
(*p*-isopropylcalix[6]arene·Cs)^+^ DMSO-AN *^b^*					
Pure AN			*c*	*c*	*c*
15.5%DMSO-84.5%AN			−18.5 ± 0.8	2.3 ± 0.4	69.8 ± 2.1
32.9%DMSO-67.1%AN			−17.2 ± 0.6	3.7 ± 0.2	70.1 ± 2.0
52.5%DMSO-47.5%AN			−15.3 ± 0.3	11.6 ± 1.7	90.3 ± 5.5
74.6%DMSO-25.4%AN			−15.2 ± 0.3	29.8 ± 5.0	151.1 ± 16.6
Pure DMSO			−16.5 ± 0.3	11.6 ± 4.5	94.0 ± 14.9

*^a^ SD* = Standard deviation; *^b^* Composition of binary mixture is expressed in mol% for solvent system; *^c^* The salt is not dissolved.

## 3. Discussion

As it can be seen from Table 1, addition of *p*-isopropylcalix[6]arene to Cs^+^ cation in all solvent systems at different temperature shows an increase in molar conductivity by increasing the ionophore concentration, which indicates that the (*p*-isopropylcalix[6]arene·Cs)^+^ complex is more mobile than free solvated Cs^+^ cation. As it is obvious from Figure 2, the slope of corresponding molar conductance (Λ*_m_*) versus ([*L*]_t_/[*M*]_t_) plots is not sharp, because the formed complex is not so much strong and since the concentrations of metal calixarene salt (MLA) are nevertheless very small and do not exceed the concentration of metal ion (M^+^), Therefore Λ*_MLA_* values are close to the values of limiting molar conductance of MLA. The molar conductivity of the complex is not reached to a limiting value at the studied mole ratio, therefore, Λ*_MLA_* cannot be determined. It is noteworthy to mentioned that, the slope changes at the point where the ionophore to cation mole ratio is about 1, which is an evidence for the formation of comparatively stable 1:1 complex between *p*-isopropylcalix[6]arene and Cs^+^ cation in these solvents.

As it can be seen from Table 2, the stability constants of (*p*-isopropylcalix[6]arene·Cs)^+^ complex increase as the temperature increases, which is an evidence for endothermic complexation reaction between the ionophore and corresponding cation. The similar behavior for all complexes indicates that stronger complexes are formed at higher temperatures.

Comparison of the stability constant values given in Table 2, reveals that for (*p*-isopropylcalix[6]arene·Cs)^+^ complex in binary solvents with higher concentration of DMSO with a relatively larger Gutmann donor number (DN:DMSO = 29.8), Cs^+^ cations were strongly solvated and hardly can be complexed by the ionophore. However, the values of stability constant increase by increasing the concentration of AN with relatively smaller Gutmann donor number (DN:AN = 14.1) in binary solvents, which is in accordance with the reverse order of their solvating ability as represented by Gutmann donor number.

It is known that the solvating ability of the solvent, as expressed by the Gutmann donor number, plays an important role in different complexation reactions. Moreover, the stability and selectivity of the formed complexes are affected by some molecular factors such as the number and character of the donor atoms in the macrocyclic ring, the polarizability and charge density of the metal ion, the nature of substituents and cavity size of the macrocyclic compound, and the character of co-anion with the cationic species [15,16].

As demonstrated in Figure 4, the changes of stability constant (log K_f_) of (*p*-isopropyl-calix[6]arene·Cs)^+^ complex with the composition of DMSO-AN binary system at different temperatures are not linear. It is possibly due to the changes happened in solvation characteristics of the ionophore, cation and even the resulting complex as the composition of the medium varied, which caused the change in the interactions of the solvents with the solutes [17].

Moreover, there are some more parameters which may affect the complexation process, such as the preferential solvation of ionophore molecules and cations as well as the changes happened in their characteristics by changing the composition of solvents medium and temperature. Preferential solvation of ions by one of the components of a mixed solvent system depends on two factors: the relative donor–acceptor abilities of the component molecules towards the ion and the interactions between solvent molecules themselves. The solvating properties of the components in mixed solvents can even be significantly modified by solvent–solvent interactions when the energy of the latter is comparable with the energy difference of solvent–ion interactions for both components [18].

According to the data which are summarized in Table 3, it can be concluded that the enthalpy and entropy values for complexation reaction between *p*-isopropylcalix[6]arene with Cs^+^ cations varied with the nature and composition of the mixed solvents [19]. This is due to variations in the extent of the contribution of such important parameters as solvation-desolvation of the species involved in the complexation reaction (*i.e.*, Cs^+^ cation, ionophore and the resulting complex). It is known that, the enthalpy and entropy of the formed complexes between macrocyclic compound and cations change with different factors such as variation in the flexibility of macrocyclic ionophore during the complexation process and the amount of cation-solvent, ionophore-solvent, complex-solvent and even solvent-solvent interactions [13,14].

The experimental values of standard enthalpy (Δ*H°_c_*) and standard entropy (Δ*S°_c_*) show that, the thermodynamic parameters for complexation reaction between *p*-isopropylcalix[6]arene and Cs^+^ cations in these solutions, did not vary monotonically with the solvent composition. A non-monotonic behavior has also been observed for thermodynamic functions of several macrocyclic compound-metal ion complex formations in some binary mixed solvents [20]. This behavior may be due to strong interactions between the constituent solvent molecules which result in changes in some of the chemical and physical properties of each of the solvents, and therefore, changing their solvating ability towards the dissolved species. In addition, the heteroselective solvation of the cation and even the macrocyclic ionophore, the character of its changes with the composition of the mixed solvent and temperature may be effective in the complexation reactions [21].

The data of Table 3 reveal that in most cases the change in Δ*H°_c_* for the complexation process is negligible, whereas the change in Δ*S°_c_* is significant. Hence, this thermodynamic quantity is the main driving force for the formation of (*p*-isopropylcalix[6]arene·Cs)^+^ complex in DMSO-AN binary solvent solutions. The changes of solvation, steric deformation of the ionophore and intermolecular ionophore-ionophore repulsions result in the change of ionophore enthalpy in complexation process. The increase in degree of freedom which caused by desolvation of cation might result in some positive entropic gain, as well as releasing the solvent molecules that involve in the interaction with ionophore. Moreover, the solvent-solvent interactions and changing in flexibility of ionophore upon complexation contribute to changes in entropy [22,23].

## 4. Experimental

### 4.1. General

*p*-Isopropylcalix[6]arene and cesium chloride were purchased from Merck and used without any further purification, except for vacuum drying over P_2_O_5_. Acetonitrile (AN) and dimethylsulfoxide (DMSO) with the highest purity available from Merck were used as solvents. 

The experimental procedures designed to obtain the formation constant of the (*p*-isopropylcalix[6]arene·Cs)^+^ complex were as follows: a solution of metal salt (5.0 × 10^−4^ M) was placed in a titration cell and the conductance of the solution was measured. In order to keep the electrolyte concentration constant during the titration, both the starting solution and the titrant had the same metal ion concentration. Then a known amount of the calixarene solution (2.5 × 10^−2^ M) was added in a stepwise manner to the titration cell using a microburette and the conductance of the resulted solution was measured after each step at the desired temperature. 

The conductance measurements were performed on a digital Cyberscan conductivity/TDS/°C/°F meter (510 CON) in a circulating water bath (Protech) with a constant temperature maintained within ±0.01 °C. The electrolytic conductance was measured using a conductivity/TDS electrode (code No: ECCONSEN91W/35608-50) which comes with stainless steel rings, cell constant of K = 1.0 cm^−1^, and a built-in temperature sensor for automatic temperature compensation (ATC).

### 4.2. Complex Formation Constant (K_f_)

The complex formation process between macrocyclic compound (ionophore) and metal ion can be expressed by the following equilibrium:

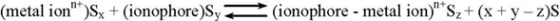

where S denotes the solvent molecule and x, y, z denote the solvation numbers of metal cation, ionophore and the resulting complex, respectively. As demonstrated, the complex formation process in solution is a multiple competition between metal ion, ionophore and solvent molecules. When the metal ion and ionophore are effectively solvated by the solvent molecules, the formation of the complex may be reduced, or even prevented. 

The complex formation of macrocyclic compounds with metal ions lowers the concentration of free metal ions in the solution. Since the mobility of free cations and complexed cations is different, the conductance of the electrolyte solution changes. Therefore, complex formation reaction can be studied by conductometry method. The following equilibrium shows a 1:1 complex formation reaction between macrocyclic compounds (*L*) with metal ion (*M^n+^*):

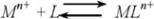
(2)


The related thermodynamic equilibrium constant is represented by:

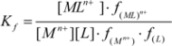
(3)
where [*ML^n+^*], [*M^n+^*] and [*L*] denote the molar concentration of the formed complex, free metal ion and free macrocyclic compounds, respectively. “ƒ” represents the activity coefficient of the existing species in the electrolyte solution.

Since the experiments are carried out under the highly dilute conditions, the activity coefficient of neutral macrocyclic compound, ƒ_(*L*)_ can be logically assumed as unity [24]. On the other hand, according to Debye-Hückel limiting law, the activity coefficients of free metal ions, ƒ_(*M*_*^n+^*_)_ and complex, ƒ_(*ML*_*^n+^*_)_ are equal to each other and their ratio is reasonably assumed as unity [25,26]. Therefore, the complex formation constant based on molar concentration is represented as below:

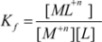
(4)


As it can be seen from Equation (5), the specific conductivity (*k*) in each point of titration process is equal to the combination of conductivity of both metal salt (*MA*) and metal calixarene salt (*MLA*). A^−^ denotes an anion:

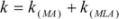
(5)


The molar conductance of metal salt before addition of macrocyclic compound, (*MA*) and molar conductance of metal calixarene salt (*MLA*) are showed in the following equations:

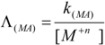
(6)

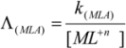
(7)
where *k_(MA)_* and *k_(MLA)_* represent the molar conductance of metal salt and metal calixarene salt. The total analytical concentration of the metal ion which is the combination of free and complexed form can be showed as:

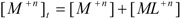
(8)


The observed molar conductance of solution during titration is described by Equation (9):

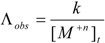
(9)


By combining the Equations (5), (6), (7) and (9) and simplifying, the equation below can be obtained:

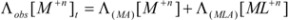
(10)

The combination of Equations (4) and (8) results in Equation (11):

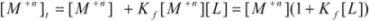
(11)

On the other hand, the total concentration of macrocyclic compound which is represented by [*L*]*_t_* can be described as:

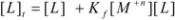
(12)

The following equation can be obtained by the substitution of Equation (4) into Equation (12):

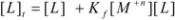
(13)

By substituting Equation (4) into Equation (10), the observed molar conductance of solution is given as below:

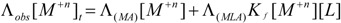
(14)

The observed molar conductance of solution can be simplified by substituting Equation (11) into Equation (14):

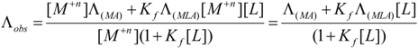
(15)

The following equation is the combination of Equations (11) and (13):

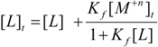
(16)

Rearranging Equation (16) yields:

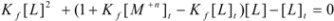
(17)

By solving the above quadratic equation, the amount of [*L*] can be found:

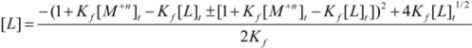
(18)

The positive root of Equation (18) represents the concentration of macrocyclic compound, [*L*] at each point of the conductometric titration. The value of complex formation constant (*K_f_*) and molar conductance of metal calixarene salt (*MLA*) can be calculated by substituting [*L*] into Equation (15).

The values of [*L*]_t_, [*M^+n^*]*_t_* and Λ*_(MA)_* which denote the total concentration of macrocyclic compound, the total analytical concentration of the metal ion and the molar conductance of metal salt before addition of macrocyclic compound, respectively, are known. The value of [*L*] can be calculated by substituting an estimated value of *K_f_* into the Equation (18). In order to obtain the amount of calculated molar conductance Λ*_cal_*, the values of *K_f_*, Λ*_(MLA)_*, Λ*_(MA)_* and [*L*] are substituted into Equation (15). This is followed by comparing the value of calculated molar conductance Λ*_cal_* and observed molar conductance (Λ*_obs_*) until the best value for complex formation constant (*K_f_*) is achieved.

### 4.3. GENPLOT Computer Program

The comparison between Λ*_cal_* and Λ*_obs_*, in order to get the least difference between them and subsequently evaluation of the best value for complex formation constant, *K_f_*, is done by using non-linear least-squares GENPLOT computer program [27], through variation of molar conductance as a function of (macrocyclic compound/metal ion) mole ratios and fitting the data into the function based on Equations (15) and (16).

The four parameters of the function are assumed as three variable parameters which are the molar conductance of metal salt Λ*_(MA)_*, the molar conductance of metal calixarene salt Λ*_(MLA)_*, complex formation constant (*K_f_*) and one non variable parameter which is the total analytical concentration of metal ion [*M^+n^*]*_t_*.

### 4.4. Enthalpy and Entropy of Complexation Process

The dependence between standard Gibbs free energy (Δ*G°_c_*) with complex formation constant (*K_f_*) and standard enthalpy (Δ*H°_c_*) and standard entropy (Δ*S°_c_*) are expressed by the following equations:

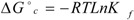
(19)

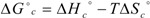
(20)
where *T* and *R* denote the absolute temperature in Kelvin and universal gas constant, respectively. By determining complex constant formation (*K_ƒ_*) in different temperatures and plotting the graph of *LnK_f_* versus *1/T* (Van’t Hoff plot), the standard enthalpy (Δ*H°_c_*) value can be found [14]:

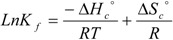
(21)

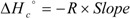
(22)

The values of Gibbs free energy (Δ*G°_c_*) and standard entropy (Δ*S°_c_*) can be obtained from Equations (19) and (20), respectively.

## 5. Conclusions

It can be concluded that the nature and composition of the solvent media can remarkably affect the thermodynamic stability, stoichiometry, selectivity and exchange kinetics of the formed complexes and the stoichiometry of the (*p*-isopropylcalix[6]arene·Cs)^+^ complex in all composition of DMSO-AN binary mixed solvents is 1:1[I:M].
